# CK1δ-Derived Peptides as Novel Tools Inhibiting the Interactions between CK1δ and APP695 to Modulate the Pathogenic Metabolism of APP

**DOI:** 10.3390/ijms22126423

**Published:** 2021-06-15

**Authors:** Aileen Roth, Fabian Gärtner, Katja Mayer, Julian Beyrle, Irina König, Uwe Knippschild, Joachim Bischof

**Affiliations:** Surgery Centre, Department of General and Visceral Surgery, Ulm University Hospital, Albert-Einstein-Allee 23, 89081 Ulm, Germany; aileen.roth@uni-ulm.de (A.R.); fabian.gaertner@uni-ulm.de (F.G.); katja-1.mayer@uni-ulm.de (K.M.); julian.beyrle@gmail.com (J.B.); irina.koenig2@hochschule-bc.de (I.K.); uwe.knippschild@uniklinik-ulm.de (U.K.)

**Keywords:** Alzheimer’s disease, AD, casein kinase 1, CK1, amyloid precursor protein, APP, therapeutic peptide, protein-peptide-interaction, amyloid-β, Aβ

## Abstract

Alzheimer’s disease (AD) is the major cause of dementia, and affected individuals suffer from severe cognitive, mental, and functional impairment. Histologically, AD brains are basically characterized by the presence of amyloid plaques and neurofibrillary tangles. Previous reports demonstrated that protein kinase CK1δ influences the metabolism of amyloid precursor protein (APP) by inducing the generation of amyloid-β (Aβ), finally contributing to the formation of amyloid plaques and neuronal cell death. We therefore considered CK1δ as a promising therapeutic target and suggested an innovative strategy for the treatment of AD based on peptide therapeutics specifically modulating the interaction between CK1δ and APP. Initially, CK1δ-derived peptides manipulating the interactions between CK1δ and APP695 were identified by interaction and phosphorylation analysis in vitro. Selected peptides subsequently proved their potential to penetrate cells without inducing cytotoxic effects. Finally, for at least two of the tested CK1δ-derived peptides, a reduction in Aβ levels and amyloid plaque formation could be successfully demonstrated in a complex cell culture model for AD. Consequently, the presented results provide new insights into the interactions of CK1δ and APP695 while also serving as a promising starting point for further development of novel and highly innovative pharmacological tools for the treatment of AD.

## 1. Introduction

Globally, approximately 50 million people are affected by dementia and due to a dramatically rising population and an increasing life expectancy, the number of people affected by dementia is expected to increase to 152 million people by 2050. With around two thirds of all cases, Alzheimer’s disease (AD) is the most common form of dementia [1]. AD is a progressive neurodegenerative disease characterized by irreversible morphological and biochemical changes in certain brain areas, in particular in the cortex and the hippocampus, which is linked to loss of cognitive function (reviewed in [2,3]). From a histological point of view, AD is mainly characterized by three pathological hallmarks including neuronal loss, extracellular amyloid plaques consisting of aggregated amyloid-β (Aβ) peptides, as well as the intracellular formation of neurofibrillary tangles composed of a hyperphosphorylated form of the microtubule-associated protein tau [2,3,4,5]. According to the amyloid cascade hypothesis, deficiencies in the processes related to production, accumulation, and disposal of Aβ are the primary cause of AD [6]. It is also believed that Aβ supports an increase in tau-phosphorylation and initiates multiple pathways leading to neuronal cell death, since Aβ oligomers show neurotoxic effects including the disruption of Ca^2+^ homeostasis, the induction of oxidative stress, excitotoxicity, inflammation, and apoptosis [2,6,7].

Aβ peptides are generated by proteolytic cleavage of the amyloid precursor protein (APP), a type-I single transmembrane protein [8,9]. APP splice variants can be found in many different tissues, whereas variant APP695 is predominantly expressed in neurons in the central nervous system. Although the physiologic function of APP remains elusive, an involvement in cell adhesion, regulation of cell–cell or cell–matrix interactions, or neuronal differentiation is under discussion [7,8,10,11]. Proteolytic cleavage of APP can either be non-amyloidogenic, resulting in generation of the neuroprotective product soluble APPα as a result of α-secretase cleavage, or amyloidogenic, leading to the formation of Aβ monomers due to cleavage mediated by β- and γ-secretase. These Aβ monomers can further aggregate into oligomers, protofibrils, and fibrils, consequently resulting in the characteristic accumulation of amyloid plaques in brain parenchyma of AD patients [10,11,12].

Increasing evidence suggests that protein phosphorylation plays a key role in mediating AD-associated pathological events. APP is phosphorylated by protein kinases at several sites, which likely affect Aβ production, for example, by modulating generation of the toxic peptide Aβ_1–42_ by β-secretase-mediated cleavage [8,13]. Several phosphorylation sites have been identified on APP that can be associated with AD. Eight potential phosphorylation sites were found in the APP cytoplasmic domain, seven of which were phosphorylated in AD brains [14]. Threonine residue 668 (T668) is phosphorylated in vivo by protein kinases glycogen synthase kinase-3β (GSK3β), stress-activated protein kinase 1b (SAPK1b)/c-Jun N-terminal kinase 3 (JNK3), cell division cycle 2 (Cdc2), and cyclin-dependent kinase 5 (Cdk5), and phospho-T668-APP was found upregulated in the hippocampus of AD brains. Inhibition of T668 phosphorylation significantly reduced Aβ production, highlighting the important role of APP phosphorylation for the Aβ-associated pathogenesis of AD [8,14]. Further evidence suggests that CK1 protein kinases (formerly termed casein kinase 1) can be involved in AD pathogenesis. The highly conserved isoforms of the CK1 family are essentially involved in the regulation of numerous cellular processes, including growth, proliferation, and differentiation as well as apoptotic processes. Alterations in CK1 expression and/or activity as well as mutations in the coding sequence of CK1 isoforms can be associated with neurological disorders and tumorigenesis [15,16]. In hippocampal regions of AD brains, CK1δ mRNA and protein levels were found upregulated by 24.4- and 33-fold, respectively [17,18]. Furthermore, CK1ε is proposed to be involved in APP processing at the γ-secretase level, since CK1ε likely regulates a component of the γ-secretase complex. Consequently, overexpression of CK1ε resulted in increased Aβ production in cells stably expressing APP695. By treating these cells with a CK1ε-specific inhibitor, this increase in Aβ production could be reduced [19]. Finally, in silico analysis revealed the presence of several potential CK1-targeted consensus motifs in the intracellular regions of APP, and phosphorylation of the ectodomain E1 of APP by CK1-like ectoprotein kinases has already been demonstrated in vitro and in cell culture [20,21]. These findings suggest that members of the CK1 family play a crucial role in AD neuropathology and highlight CK1 as a promising target for the development of new therapeutics for the treatment of neurodegenerative diseases.

Although CK1-specific small molecule inhibitors (SMIs) have already been shown to reduce Aβ production, they usually inhibit the activity of certain CK1 isoforms in general. Thereby, these inhibitors induce certain side effects due to inhibition of CK1 isoforms in essential cellular processes and in healthy tissue. To circumvent this, alternatives to SMIs focusing on intervention in CK1 activity at the level of kinase–substrate interactions are of special interest. Therefore, in the present study, we aimed at characterizing CK1δ-derived peptides that specifically block the interaction of CK1 isoform δ and APP695 without directly affecting kinase activity. A CK1δ-derived peptide library was first tested for interactions with APP695 protein fragments and, subsequently, competitive inhibition of CK1δ-mediated phosphorylation of APP695 fragments by interacting peptides was analyzed. Finally, cell entry of selected peptides and peptide-mediated effects on Aβ production and formation of amyloid plaques was investigated in an established cell culture model for AD. In summary, our results contribute to the detailed characterization of the interactions of CK1δ and APP695 and highlight a new therapeutic option for AD.

## 2. Results

### 2.1. CK1δ-Derived Peptides Bind to APP695 Fragments and Inhibit Their Phosphorylation by CK1δ

**Figure 1:**
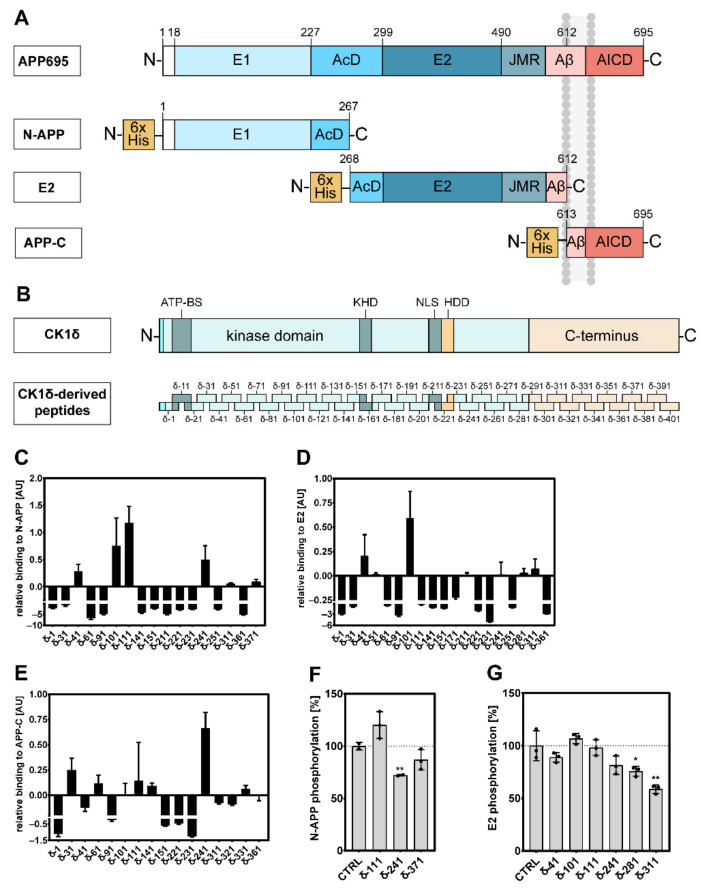
CK1δ-derived peptides interact with APP695 fragments N-APP, E2, and APP-C and inhibit phosphorylation of N-APP and E2. (**A**) Domain structure of APP695 and the subsequently used protein fragments N-APP, E2, and APP-C [22]. (**B**) Domain structure of CK1δ and localization of CK1δ-derived peptides within in the full-length protein sequence. (**C,D,E**) Detection of CK1δ-derived peptides specifically binding to APP695 fragments N-APP, E2, and APP-C was performed by ELISA (*n* = 3). Specific binding to wells coated with APP695 fragments is demonstrated relative to unspecific binding to uncoated wells. (**F,G**) Effects of CK1δ-derived peptides on CK1δ-mediated phosphorylation of APP695 fragments N-APP and E2 were determined by performing in vitro kinase assays in the absence (DMSO; CTRL) or presence of 20 µM of the indicated CK1δ-derived peptides using GST-CK1δ as kinase (*n* = 3). Statistical analysis was performed using one-tailed *t*-test shown as * for *p* ≤ 0.05 or ** for *p* ≤ 0.01. AcD: acidic domain; AICD: APP intracellular domain; APP: amyloid precursor protein; ATP-BS: ATP binding site; AU: arbitrary units; CTRL: control; E1/E2: ectodomain 1/2; HDD: homodimerization domain; JMR: juxtamembrane region; KHD: kinesin homology domain; NLS: nuclear localization signal; δ: CK1δ-derived peptide.

Previous studies already demonstrated the importance of protein kinases, including CK1δ, for the pathogenesis of AD by modulating APP metabolism and enhancing Aβ production [14,20]. With the aim to perform peptide–protein interaction analysis as well as phosphorylation analysis, the protein sequence of full-length APP695 was split into smaller protein fragments (further referred to as N-APP, E2, and APP-C; Figure 1A), thereby facilitating production of the respective fragments as recombinant proteins.

In order to identify CK1δ-derived peptides interacting with APP695, ELISA interaction analysis was performed using the CK1δ-derived peptide library introduced in the Materials and Methods section and depicted in Figure 1B (for a more detailed presentation see Supplementary Figure S1). In initial screening approaches, peptides interacting with APP695 fragments N-APP (amino acids 1 to 267 of APP695), E2 (amino acids 268 to 612 of APP695), and APP-C (amino acids 494 to 695 of APP695) were identified (Supplementary Figure S2). Confirming this initial data, CK1δ-derived peptides showing significant binding to the tested fragments of APP695 were further tested for specificity of interaction by comparing their binding to APP695 fragment-coated to uncoated wells (Figure 1C–E). Peptides, which demonstrated to have a higher affinity to the plastic surface of uncoated wells than to wells coated with APP695 protein fragments, are not shown in Figure 1.

According to the comparison of N-APP-coated to uncoated wells, peptides δ-41, δ-101, δ-111, and δ-241 were able to specifically interact with N-APP (Figure 1C). For the comparison of E2-coated to uncoated wells peptides δ-41, δ-101, and δ-311 were shown to achieve the most significant differences (Figure 1D), and when determining specific binding to APP-C-coated wells, peptides δ-31, δ-61, δ-141, and δ-241 were identified as peptides demonstrating the most significant binding (Figure 1E). For all other tested peptides, binding to APP695 fragment-coated wells was either determined to be unspecific or not significant. In summary, considering data obtained for all APP695 protein fragments, most significant binding could be demonstrated for peptides δ-41, δ-101, and δ-241.

With the aim to confirm the interactions of CK1δ-derived peptides with the tested APP695 fragments and to demonstrate the competitive phosphorylation-inhibitory potential of specifically binding peptides, in vitro kinase assays were performed. Therefore, APP695 fragments N-APP, E2, or APP-C were used as substrates, and selected CK1δ-derived peptides were used as potential inhibitors competing with CK1δ for the binding to APP695 fragments and thus blocking the phosphorylation of these fragments by recombinant GST-CK1δ. Initially performed in vitro kinase assays demonstrated robust phosphorylation of N-APP and E2 by GST-CK1δ. In contrast, no or only very weak CK1δ-mediated phosphorylation could be detected for APP695 protein fragment APP-C. In all phosphorylation reactions, autophosphorylation of CK1δ was clearly detectable, thereby indicating that the used kinase was active and that reaction conditions were appropriate (Supplementary Figure S3).

Subsequently, in vitro kinase assays using N-APP or E2 as substrates were performed in the absence (DMSO) or presence of 20 µM of the indicated CK1δ-derived peptides. Significant inhibition of CK1δ-mediated phosphorylation of N-APP could be detected for peptide δ-241 (Figure 1F), while phosphorylation of E2 was significantly reduced in the presence of peptides δ-281 and δ-311 (Figure 1G). Inhibitory effects of the other interacting peptides δ-371 (for phosphorylation of N-APP) and δ-41 (for phosphorylation of E2) were less remarkable, and no effects at all could be observed for peptides δ-101 and δ-111 (Figure 1F,G). Control reactions demonstrating that inhibition of CK1δ-mediated phosphorylation of N-APP and E2 is substrate-specific were conducted using the prototypic substrate α-casein. Except for peptide δ-311, phosphorylation of α-casein by CK1δ was not inhibited by any of the tested peptides, indicating that inhibitory effects are specific for N-APP or E2 (Supplementary Figure S4). Due to weak or even non-existent phosphorylation of APP-C, inhibitory effects of CK1δ-derived peptides have not been investigated for this domain of APP695.

### 2.2. In Silico Simulations Confirm Interactions of CK1δ-Derived Peptides with APP Protein Fragments

As a means to further evaluate whether the identified binding and phosphorylation-inhibiting peptides actually originate from potential interacting domains, surface maps of CK1δ were generated highlighting the localization of identified peptides in the full-length protein. Therefore, the localization of the sequences of peptides δ-41, δ-101, δ-111, δ-241, and δ-281 was displayed in the cartoon and surface representation of the 3D structure of CK1δ (PDB entry 6GZM [23]). Due to its intrinsically disordered structure, the CK1 C-terminal domain cannot be illustrated by using this or any other available protein structure data. Therefore, localization of peptides δ-311 and δ-371, being located within this C-terminal domain, could not be demonstrated.

**Figure 2:**
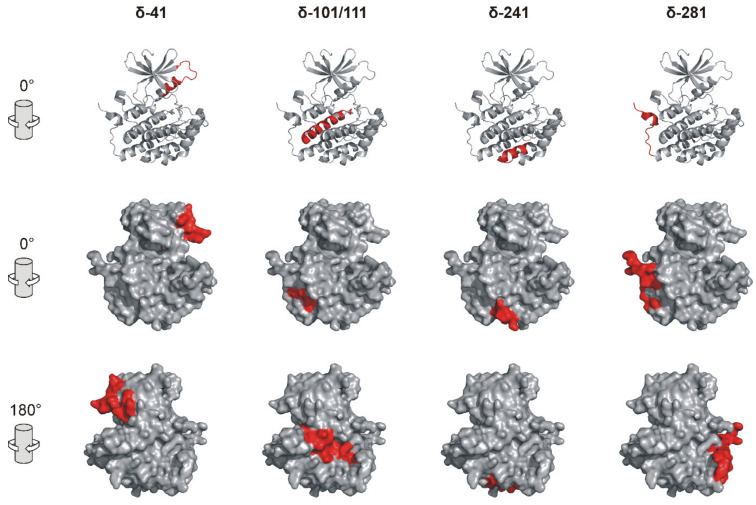
Localization of selected CK1δ-derived peptides in CK1δ protein structure. Cartoon and surface representation of the molecular structure of truncated CK1δ (encompassing amino acids 1 to 295; PDB entry 6GZM) showing the localization of the CK1δ-derived peptides δ-41, δ-101/111 (these directly adjacent peptides are shown in the same image), δ-241, and δ-281 in CK1δ. Sequences of CK1δ-derived peptides are highlighted in red. Peptides δ-311 and δ-371 cannot be displayed because structural data of the CK1δ C-terminal domain has not been available. Figures were generated by using the protein structure visualization software PyMOL (Schroedinger). Surface images are displayed in original view (0°) and after rotation by 180°.

Most parts of the displayed peptides were well exposed to the protein surface of the CK1δ protein structure (Figure 2). Therefore, these sequence patches in CK1δ could also be involved in mediating the binding of full-length CK1δ to interaction partners like APP695 under physiologic in vivo conditions.

**Figure 3:**
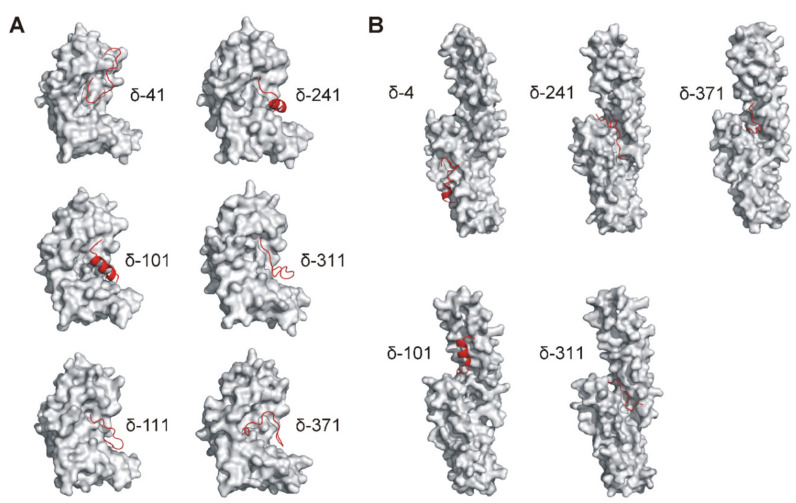
Peptide–protein docking models simulating interactions of CK1δ-derived peptides with N-APP/E1 and E2 protein domains of APP. CK1δ-derived peptides previously identified to bind and inhibit phosphorylation of APP695 protein fragments were used for in silico docking experiments using a protein structure encompassing (**A**) the N-APP/E1 domain (4PWQ [25]) or (**B**) the E2 domain of APP (3UMI [26]). Docking simulation was performed by CABS-dock server for flexible protein–peptide docking, and the best-ranked model (according to cluster density) for each docking analysis is displayed. Peptide structures are shown in red.

Previously performed in vitro analyses already identified several peptides able to interact with the N-APP and/or E2 protein domains of APP695. With the aim to support this in vitro data the fully flexible in silico peptide–protein docking tool CABS-dock [24] was used to simulate the interactions between the previously identified binding and phosphorylation-inhibiting peptides with the N-APP domain (best represented by E1 structural data deposited under PDB entry 4PWQ [25], further referred to as N-APP/E1) and/or the E2 domain (best represented by PDB entry 3UMI [26]) of APP695. The top-ranked docking prediction models presented in Figure 3 exhibit the highest cluster densities and therefore represent the most probable docking states of the indicated peptides with N-APP/E1 or E2.

Most of the docked peptides occupied parts of a cavity formed between the N- and C-terminal proportions of the N-APP/E1 protein structure (Figure 3A and Supplementary Figure S5). Only peptide δ-41 was predicted to predominantly bind the N-terminal part of N-APP/E1 comprising the growth-factor-like domain as well as one of the heparin-binding domains of APP [25]. Peptides δ-101 and δ-241 are supposed to contain helical features. Superior docking, as indicated by ligand-RMSD values (root mean square deviation values as a differentiation measure between cluster elements) below 5 Å, was simulated for all peptides except for δ-41 (Supplementary Table S1).

When docking to the E2 protein structure was performed, sites of interaction with peptides δ-241, δ-281, and δ-311 were located close to the cavity formed between the N- and C-terminal helical domains of E2 (Figure 3B and Supplementary Figure S6). In contrast, peptide δ-41 was docked to the C-terminal domain close to the collagen-binding domain of APP [27] and peptide δ-101 was docked to the N-terminal domain. For these two peptides, a β-sheet or an α-helical structure, respectively, was assumed. Superior ligand-RMSD values below 5 Å were calculated by the docking algorithm for all peptides except for δ-41. Best values for overall cluster density and average ligand-RMSD could be determined for peptide δ-241 (Supplementary Table S1).

Prior to each docking approach, the secondary structural features of each peptide were calculated by the PSIPRED algorithm [28,29]. When comparing structural features of the docked peptides with features present in CK1δ protein (using PDB entry 6GZM as a reference [23]), only peptide δ-241 bound to N-APP/E1, δ-41 bound to E2, as well as δ-101 bound to both N-APP/E1 and E2 appeared to share similar structural features (see Figure 2 (CK1δ), Figure 3 (N-APP/E1 and E2), and Supplementary Table S1 for comparison).

### 2.3. CK1δ-Derived Peptides Successfully Enter Neuronal Cells without Inducing Cytotoxic Effects

Having established the potential of several CK1δ-derived peptides to block the interactions between CK1δ and APP by biochemical approaches in vitro, the identified peptides subsequently had to demonstrate cell-penetrating properties and therapeutic potential. Initially, cytotoxic effects of selected peptides were tested by using transduced human neural progenitor cells (hNPCs) (see Section 2.4 for further information), which were differentiated to neurons and glial cells for six days before being treated with CK1δ-derived peptides in a defined dilution series for 24 h. For all tested peptides, no unspecific cytotoxic effects could be observed in the analyzed concentration range between 0.1 and 10 µM (data not shown). With the aim to maintain the peptides’ therapeutic potential while keeping peptide concentration as low as possible, a concentration of 1 µM was selected for subsequent treatments.

**Figure 4:**
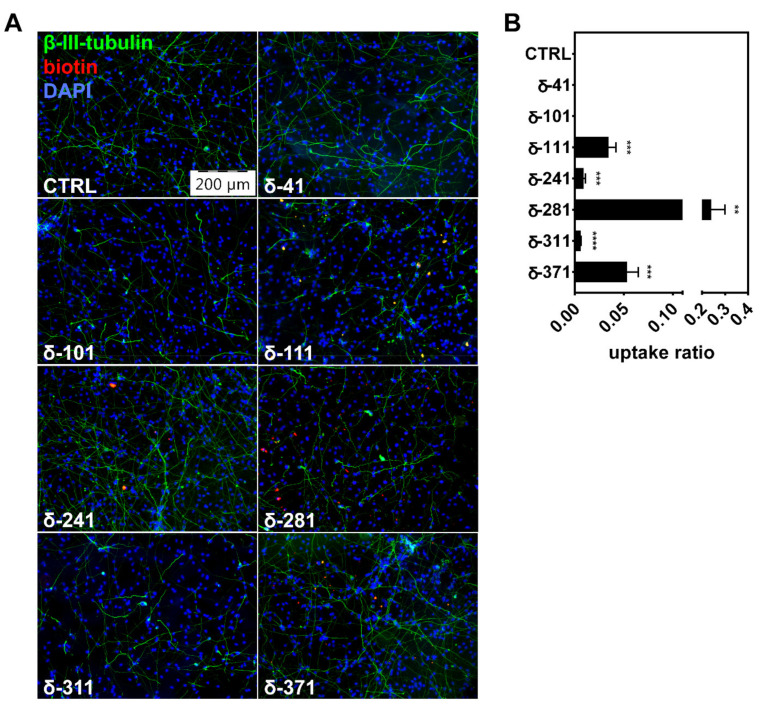
CK1δ-derived peptides are able to enter living cells. (**A**) Differentiated naïve hNPCs were treated with 1 µM of the indicated biotinylated CK1δ-derived peptides for 24 h. Cells were fixed and stained with DAPI (blue, nuclei), anti-β-III-tubulin (green, neuronal microtubule network), and streptavidin-TRITC (red, biotinylated peptides). Images were taken at 10× magnification using an epifluorescence microscope (Olympus IX81). Scale bar: 200 µm. (**B**) Quantification of peptide uptake was done by cell counting and calculated as the number of detected peptide accumulations per cell (*n* = 3). Statistical analysis was performed using one-tailed *t*-test shown as ** for *p* ≤ 0.01, *** for *p* ≤ 0.001, or **** for *p* ≤ 0.0001.

Prior to the determination of peptide-mediated specific effects on cellular AD-associated functions (like APP metabolism), cell entry was validated in differentiated naïve hNPCs being treated with CK1δ-derived peptides (1 µM) for 24 h. Afterwards, cells were fixed and stained using DAPI (for staining of nuclei) and an antibody specific to β-III-tubulin (for staining the neuron-specific microtubule network). The biotinylated CK1δ-derived peptides were stained using streptavidin-TRITC, and cell entry of peptides into differentiated hNPCs was quantified. Especially for peptides δ-111, δ-281, and δ-371, strong intracellular staining could be observed, indicating that these peptides successfully penetrated the cell membrane and entered the cells (Figure 4A,B). The highest ratio for peptide uptake could be calculated for δ-281, demonstrating entry to approximately 24% of all analyzed cells (uptake ratio of 0.24). Peptide-specific staining was rather weak for δ-241 and δ-311, whereas no staining at all could be observed for δ-41 and δ-101.

### 2.4. Treatment with CK1δ-Derived Peptides Results in Lower Aβ Levels and Reduced Formation of Amyloid Plaques

Investigation of the therapeutic effects of CK1δ-derived peptides was performed using the Alzheimer’s-in-a-dish model published by Kim and colleagues, which is based on hNPCs virally transduced with AD-associated mutant forms of APP695 (Swedish and London mutation) and presenilin 1 (PSEN1) with the deletion of exon 9 (ΔE9) [30]. Transduced cells usually secrete high levels of Aβ, and, finally, the formation of amyloid plaques can be detected in 3D cultures after at least six weeks of cell culture maintenance [30].

With the aim to analyze the therapeutic potential of selected CK1δ-derived peptides, 3D cultures of transduced hNPCs were established, and, following differentiation for six days, cells were treated with 1 µM of the indicated CK1δ-derived peptides for a total period of six weeks. Cell viability of thick-layer 3D cultures was routinely monitored by lactate dehydrogenase (LDH) release assay, which detected no substantial alterations in cell viability as consequences of treatment with the indicated peptides. Increased LDH release could only be detected after the first two days of treatment and might be due to effects related to changes in media composition, since the addition of solvent alone results in a similarly increased release of LDH from control cells. Additionally, control treatment with β-secretase inhibitor IV at ten times higher concentration compared to peptide treatment (10 µM [31] for β-secretase inhibitor IV vs. 1 µM for CK1δ-derived peptides) was not associated with considerable effects on LDH release during the entire observation period (Supplementary Figure S7).

**Figure 5:**
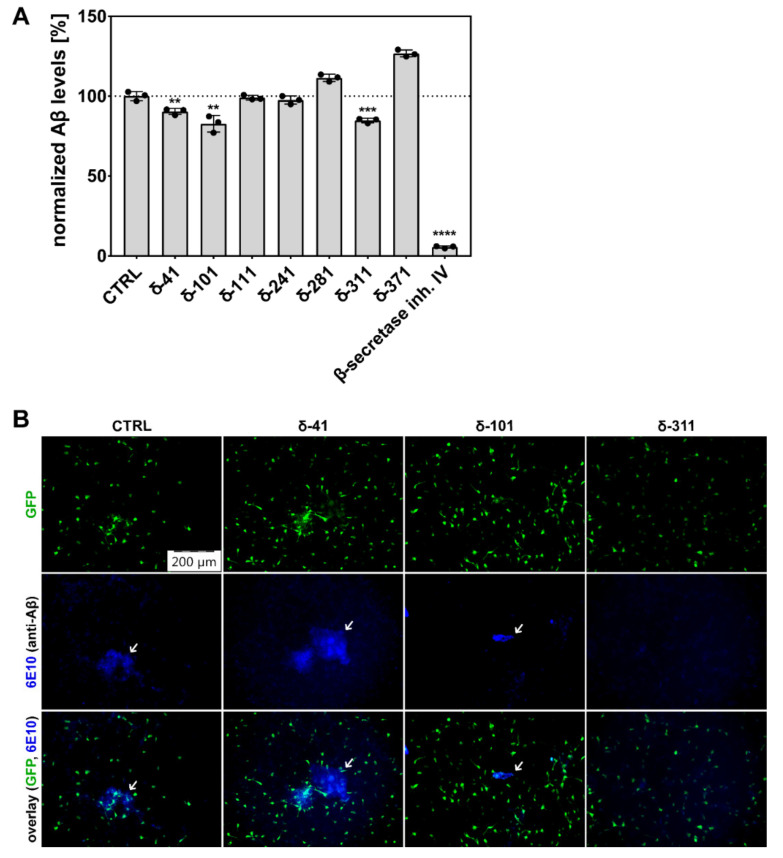
Generation of Aβ and formation of amyloid plaques was reduced by treatment with CK1δ-derived peptides. (**A**) Thick-layer 3D cultures of transduced and differentiated hNPCs were treated with DMSO (CTRL), 1 µM of the indicated CK1δ-derived peptides, or 10 µM of β-secretase inhibitor IV for six weeks. Levels of Aβ in conditioned media were determined by ELISA. Results were normalized towards Aβ levels detected for control cells. Statistical analysis was performed using one-tailed *t*-test shown as ** for *p* ≤ 0.01, *** for *p* ≤ 0.001, or **** for p ≤ 0.0001. (**B**) Thin-layer 3D cultures of transduced and differentiated hNPCs were fixed and stained after being treated with DMSO (CTRL) or 1 µM of the indicated CK1δ-derived peptides for six weeks. GFP fluorescence results from transduction with the lentiviral construct coding for mutant APP695 (Swedish and London mutation) and GFP. Amyloid plaques were stained using an Aβ-specific antibody. Images were taken at 10× magnification using an epifluorescence microscope (Olympus IX81) (*n* = 3). Scale bar: 200 µm.

After six weeks of constant 3D culture and treatment with CK1δ-derived peptides or specific SMIs, levels of secreted Aβ were determined by ELISA using conditioned media from thick-layer 3D cultures. Interestingly, Aβ levels secreted by cells treated with peptides δ-41, δ-101, and δ-311 were significantly reduced in comparison to untreated controls (Figure 5A). Aβ levels were reduced by approximately 94% by β-secretase inhibitor IV, which was used as positive control. Further supporting these results, amyloid plaques formed from secreted Aβ were detected in thin-layer 3D cultures by immunofluorescence staining. Staining with an Aβ-specific antibody revealed that amyloid plaques can still be found in cell cultures treated with peptide δ-41, whereas plaques were substantially less frequent and smaller in size for cell cultures treated with δ-101 or δ-311 (Figure 5B).

## 3. Discussion

Previous studies provided extensive evidence that members of the CK1 family might be involved in the development of AD by modulating APP metabolism and favoring Aβ production [8,14,17,19,21]. Consequently, CK1 represents a potential target molecule for the treatment of AD. As an alternative to SMIs in the present study, we investigated a novel therapeutic strategy based on the use of CK1δ-derived peptides. These peptide therapeutics used to target kinase-mediated functions are supposed to interfere with the actual interaction motif for the kinase, which is present in the substrate. Consequently, the ideal peptide only affects kinase activity towards this specific target [32]. In the present study, we demonstrated that CK1δ-derived peptides specifically block the interaction between CK1δ and APP in vitro and show significant therapeutic effects in a cell culture model for AD.

ELISA-based interaction analysis was performed to define the dominant motifs involved in mediating the interaction of CK1δ with APP695 and identified most significant binding to APP695 protein fragments N-APP, E2, and APP-C for a set of seven peptides (δ-41, δ-101, δ-111, δ-241, δ-281, δ-311, and δ-371). The interaction potential of these peptides as well as the potential to competitively block the interaction of CK1δ with APP695 has been further analyzed by in vitro phosphorylation experiments. Among the set of seven CK1δ-derived peptides identified in ELISA, only three peptides were able to significantly reduce phosphorylation of N-APP or E2. However, these peptide-mediated effects are not as substantial as those being induced by SMIs and failed to reduce phosphorylation by more than 41% (as observed for peptide δ-311 and phosphorylation of E2). With IC_50_ values within the submicromolar range, most current CK1-specific SMIs would show more potent effects on CK1δ than was observed for the CK1δ-derived peptides at the selected concentration [15]. Nevertheless, the major advantage of the tested peptides was the highly substrate-selective inhibition of N-APP and E2 phosphorylation, since no inhibition of CK1δ-mediated phosphorylation of α-casein could be observed for most peptides. This superior selectivity of CK1δ-derived peptides has already been pointed out in a previous study analyzing the inhibition of CK1δ-mediated phosphorylation of α-tubulin [33].

For the C-terminally located APP695 fragment APP-C, containing the transmembrane domain (TMD) as well as the intracellular domains (AICD) of APP695, only weak phosphorylation by CK1δ could be observed. Although APP695 in general can be phosphorylated by CK1δ and ectoprotein-CK1 during biosynthesis and trafficking, its TMD is buried within the phospholipid bilayer and can probably not be accessed by protein kinases [11,20]. Additionally, when working with recombinant proteins, the highly hydrophobic TMD is likely masked by hydrophobic molecules, which need to be included in the purification procedure to facilitate isolation of soluble protein but might also block in vitro phosphorylation by CK1 [34]. Either way, CK1-mediated phosphorylation of the TMD-related proportion of APP695 appears not to be relevant, as can be concluded from the performed biochemical analysis. Vice versa, these results demonstrate that major sites for CK1δ-mediated phosphorylation are located within the N-APP and E2 fragments of APP695. This finding is further confirmed by a ScanSite-based search for phosphorylation motifs, demonstrating that the majority of putative CK1-targeted phosphorylation sites are located in the acidic domain within amino acids 266 to 295 (data not shown) as well as by a previous report localizing phosphorylation sites targeted by CK1 within amino acids 181 to 224 of APP695 [20,35].

The respective sequences of CK1δ-derived peptides interacting with APP695 are generally expected to derive from the protein surface of CK1δ, thereby resembling actual kinase–substrate interaction motifs. However, while most of the identified interacting peptide sequences are localized on the CK1δ protein surface, peptide δ-241 especially occupies only a rather small surface area while still demonstrating remarkable binding to N-APP and APP-C. Consequently, the size of the corresponding surface area of each peptide on CK1δ does not necessarily correlate with the results of ELISA, indicating that the binding affinity of each peptide in general is more important for its specific interaction with the tested APP695 fragments. Furthermore, peptide sequences presenting robust interaction with APP695 fragments may also be located within domains distant to the active site of the kinase. This is supported by the fact that binding motifs for CK1 might be different to CK1-targeted phosphorylation motifs, which also can be distant to each other within substrate proteins [36].

In addition to the above-mentioned issues, the assumption that isolated CK1δ-derived peptides statically remain in shape as present in the whole protein structure of CK1δ might be generally misleading. While the native tertiary structure as well as the stability of the full-length protein is based on the sum of interactions determined by its constituting amino acids (e.g., hydrophobic effects, polarities, charges, stabilizing disulphide bridges, etc.), the structure and shape of isolated peptides is rather flexible and thus might not closely reproduce the actual interaction interface of both full-length proteins as present in vivo [37]. Prior to the performed peptide–protein docking simulations, the secondary structures of all analyzed peptides have been predicted by the PSIPRED algorithm [28]. However, only for peptides δ-41 (docking to E2), δ-101 (docking to N-APP/E1 and E2), and δ-241 (docking to N-APP/E1), the structural features presented by the corresponding sequence in full-length CK1δ could also be reproduced by the isolated peptide sequence in the best-ranked docking simulations. In most cases, the structural features of full-length CK1δ are not represented by the isolated peptides when docking to N-APP/E1 or E2. As a further limitation, all docking simulations had to be performed using structural data, which were available for fragments of APP695 only. Even though these models suggest that not all of the tested CK1δ-derived peptides resemble the situation presented by full-length CK1δ and APP695 in vivo, the superior evaluation of these simulations (as indicated by the obtained parameters used for ranking) still clearly supports our in vitro data demonstrating significant peptide–protein interaction with and competitive inhibition of the CK1δ-mediated phosphorylation of APP695 fragments.

Although all presented in vitro data was generated by using artificial fragments of APP695, obtained results were successfully complemented by the effects observed in subsequently performed cell culture experiments. Prior to investigation of therapeutic effects on AD-like phenotypes, peptide-induced cytotoxic effects and cell entry of CK1δ-derived peptides were tested using differentiated hNPCs. Consistent with the highly substrate-selective effects of CK1δ-derived peptides detected by in vitro kinase reactions, no cytotoxic effects on differentiated hNPCs could be observed. This finding indicates that no general inhibition of CK1δ kinase activity was induced by treatment with the selected CK1δ-derived peptides. In general, cell entry into differentiated hNPCs could be observed for most but not all tested peptides. The plasma membrane represents the final barrier through which therapeutic agents must penetrate to enter the cell, and the permeability of hydrophilic, small-molecular drugs is usually poor. Because uptake mechanisms like simple or facilitated diffusion, active transport, or even endocytosis are often insufficient, external delivery systems such as cell-penetrating peptides (CPPs), antibodies, or liposomes might be used to improve delivery and cell uptake of peptides [38,39,40]. However, at least for peptides δ-111, δ-281, and δ-371 used in our experimental approach, extensive cell entry into differentiated hNPCs could be observed without applying any further modifications on peptide sequence or delivery.

Finally, therapeutic effects of CK1δ-derived peptides were demonstrated using the Alzheimer’s-in-a-dish model, which can be used to recapitulate AD-like phenotypes including Aβ and tau pathology based on a 3D human neural cell culture system [30,41]. In order to establish this system, an immortalized hNPC cell line is transduced with lentiviral vectors encoding mutated APP695 (Swedish and London mutation) and mutated PSEN1 (ΔE9), which are both associated with familial and early-onset forms of AD. Transduced hNPCs expressing high levels of mutant APP695 and PSEN1 are then seeded in 3D culture and differentiated by growth-factor deprivation. This 3D culture system is superior to conventional 2D systems as it closely mimics the in vivo environment and supports neuronal differentiation as well as the formation of neuronal networks. Using this cell culture model, extracellular aggregation of Aβ can be detected after six weeks of differentiation, whereas robust tau phosphorylation in neurites and cell bodies as well as tau pathology in general can be observed after 10 to 14 weeks (summarized in [30,41]).

Because extracellular Aβ aggregates in the selected cell culture model become obvious after six weeks of differentiation, the established 3D cultures were treated for six weeks with selected CK1δ-derived peptides as well as a β-secretase-specific SMI serving as control. For peptide δ-311, moderate levels of cell entry were detected, and, subsequently, superior effects on the generation of Aβ and amyloid plaque formation could be demonstrated. Interestingly however, reduction in Aβ levels were not necessarily correlated with the results of the cell entry analysis. Although cell penetration was most obvious for peptides δ-111, δ-281, and δ-371, Aβ levels were significantly reduced after treatment with δ-41, δ-101, and δ-311. Since peptides in general are prone to rapid degradation and no peptide-specific staining could be observed for peptides δ-41 and δ-101 although both peptides demonstrate significant effects on APP metabolism, these peptides might be well taken up into the cells but are then quickly metabolized [42]. Alternatively, the biotin tag of these peptides could be masked by binding to APP, resulting in reduced or even absent staining with TRITC-labeled streptavidin. In the case of δ-41, the peptide-mediated effects on Aβ levels and amyloid plaque formation were rather low, whereas the effects of δ-101 were sufficient to induce a substantial reduction in Aβ levels and plaque formation. However, Aβ levels were only reduced by a maximum of 13% as observed for peptide δ-101. We assumed that the effects on Aβ levels could be potentiated by combined treatment with two or more CK1δ-derived peptides, since each peptide potentially occupies a different interaction site for CK1δ on the APP695 protein. Therefore, effects mediated by concurrent treatment with different CK1δ-derived peptides (e.g., a combination of δ-101 and δ-311) could result in more significant additive or synergistic effects.

In summary, CK1δ-derived peptide δ-101 represents an especially potent therapeutically active peptide, which displayed superior binding to APP695 protein fragments, and has the unique potential to preserve its native and stable conformation (compared to full-length CK1δ) when interacting with APP. Together with other promising candidate peptides like δ-311, it might serve as a starting point for the development of future strategies for the treatment of AD.

## 4. Materials and Methods

### 4.1. Peptide Library

The peptide library was generated according to the amino acid sequence of human CK1δ (transcription variant 1, UniProt entry P48730) (Supplementary Figure S1 and Figure 1B). Each CK1δ-derived peptide consisted of 15 amino acids in length with five amino acids at each end overlapping with the previous and the next peptides. Additionally, each peptide was tagged with biotin at the N-terminal end separated from the CK1δ-specific sequence by a SGSG-spacer. Peptides were produced by Dr. Hubert Kalbacher (Interfaculty Institute of Biochemistry, Tübingen University, Germany) as described previously [33] and Biomatik (Toronto, ON, Canada). All peptides were > 95% pure as determined by analytical reversed-phase chromatography (RP-HPLC) and were dissolved in DMSO.

### 4.2. Plasmid Vector Constructs for Protein Expression

The codon-optimized bacterial expression plasmid pET28a(+)APP695 encoding for N-terminal 6xHis-tagged wildtype human APP695 was synthesized by Biomatik (Toronto, ON, Canada). To generate the plasmid pET28a(+)N-APP encoding for the N-terminal APP695 fragment N-APP (amino acids 1 to 267 of APP695), the amino acid at 268 was substituted to the stop codon TAA. The plasmid pET28a(+)E2 encoding for the APP695 fragment E2 (amino acids 268 to 612) was generated in two steps by deleting amino acids 1 to 267 and substituting amino acid 613 to the stop codon TAA. The third plasmid, pET28a(+)APP-C encoding for the C-terminal APP695 fragment APP-C (amino acids 494 to 695), was produced by deletion of amino acids 1 to 493. The primer sets used for site-directed mutagenesis by inverse PCR-based were: 5‘-AGCAACCGAATAAACCACCAGCATTG-3′ and 5‘-TCTTCATACGGTTCTTCTG-3′ for N-APP, 5‘-TCATCAGAAATAAGTTTTCTTTGCAGAAGATGTTGG-3′ and 5‘-TGCACTTCATAACCGCTATC-3′ as well as 5‘-CGTACCACCAGCATTGCAA-3′ and 5‘-GGATCCGCGACCCATTTG-3′ for E2, and 5′-GAACAGAATTATAGTGATGATGTGC-3′ and 5‘-GGATCCGCGACCCATTTG-3′ for APP-C (Biomers.net, Ulm, Germany). Successful introduction of deletions and mutations was confirmed by Sanger DNA sequencing (Eurofins Genomics, Munich, Germany).

### 4.3. Recombinant Protein Expression and Purification

Protein production of GST-tagged CK1δ in *Escherichia coli* SoluBL21^TM^ (Genlantis, San Diego, CA, USA) was induced with 0.5 mM IPTG at an OD_600_ of 0.6 AU and proteins were expressed at 18 °C for 18 h. Afterwards, bacteria cultures were harvested by centrifugation and lysed in lysis buffer containing 20 mM Tris-HCl (pH 7.6), 150 mM NaCl, 0.5% (*v/v*) NP-40, 10% (*v/v*) glycerol, 1 mM EDTA, 1 mM EGTA, 1 mM benzamidine, 0.25 µg/mL aprotinin, and 1 mM DTT. The lysate was cleared by centrifugation and incubated with 300 µL of glutathione sepharose medium (50% (*v/v*) Glutathione Sepharose^®^ 4 Fast Flow (Cytiva, Freiburg, Germany) in PBS) at 4 °C for 2 h. The mixture was centrifuged and washed three times in lysis buffer containing 300 mM NaCl and twice in 20 mM Tris-HCl (pH 7.6) containing 50 mM NaCl, 0.25 µg/mL aprotinin, and 1 mM EDTA. The bound protein was eluted with elution buffer, which was composed of 50 mM Tris-HCl (pH 7.6), 1 mM EDTA, and 5 mM reduced glutathione. For bacterial expression of APP695 fragments, the pET28a(+) expression plasmids were transformed into *Escherichia coli* SHuffle^®^ T7 Express (New England Biolabs, Ipswich, MA, USA). Gene expression was induced with 1 mM IPTG at an OD_600_ of 0.6 AU, and incubation continued for an additional 2.5 h at 37 °C. Bacteria cultures expressing 6xHis-tagged N-APP or E2 were harvested by centrifugation and lysed in lysis buffer consisting of 50 mM sodium phosphate buffer (pH 8.0), 150 mM NaCl, 10 mM imidazole, 1 mM benzamidine, and 0.25 µg/mL aprotinin. Lysates were cleared by high-speed centrifugation, filtered, and loaded onto equilibrated cOmplete His-Tag purification columns (Roche, Mannheim, Germany). Columns were washed with washing buffer (50 mM sodium phosphate buffer (pH 8.0), 1 M NaCl, 10 mM imidazole, 1 mM benzamidine, 0.25 µg/mL aprotinin) and eluted with elution buffer (50 mM sodium phosphate buffer (pH 8.0), 500 mM imidazole, 0.25 µg/mL aprotinin). For bacteria expressing APP-C, lysis buffer was additionally supplemented with 0.1% (*w/v*) SDS and 0.5% (*v/v*) glycerol. The lysate was cleared by high-speed centrifugation, and the pellet was washed twice in 50 mM sodium phosphate buffer (pH 8.0), 2 M urea, 2% (*v/v*) Triton-X 100, 0.1% (*w/v*) SDS, and 0.5% (*v/v*) glycerol followed by a third washing step in 50 mM sodium phosphate buffer (pH 8.0), 0.1% (*w/v*) SDS, and 0.5% (*v/v*) glycerol. Afterwards, the insoluble protein was solubilized and boiled in urea buffer containing 50 mM sodium phosphate buffer (pH 8.0), 8 M urea, 1 mM NaCl, 10 mM imidazole, 1 mM benzamidine, 0.25 µg/mL aprotinin, 0.1% (*w/v*) SDS, and 0.5% (*v/v*) glycerol followed by centrifugation. The cleared lysate was filtered and loaded onto an equilibrated cOmplete His-Tag purification column. Protein refolding was performed with a gradient from urea buffer to urea-free lysis buffer within 960 min. Protein was eluted as mentioned above. The purified APP695 fragments were dialyzed and concentrated using Amicon Ultra-0.5 mL centrifugal filters (Merck Millipore, Darmstadt, Germany). All proteins were stored in 10% (*v/v*) glycerol at −80 °C after snap freezing in liquid nitrogen.

### 4.4. Direct-Binding ELISA

APP695 protein fragments were coated onto 96-well Nunc MaxiSorp plates (Thermo Fisher Scientific Inc., Waltham, MA, USA) at a final concentration of 10 ng/µL in 100 mM carbonate buffer (pH 9.6) at 4 °C overnight. In order to avoid unspecific binding, protein fragments were blocked with 5% FCS in PBS. After brief washing with 0.05% Tween in PBS, 1 µg of biotinylated CK1δ-derived peptides were added and incubated at RT for 2.5 h. DMSO was used as a negative control. Protein–peptide complexes were washed with 0.05% Tween in PBS and incubated with HRP-streptavidin (Thermo Fisher Scientific Inc., Waltham, MA, USA) at a dilution of 1:8000 in 0.5% FCS in PBS at RT for 2 h. After another washing step, plates were incubated with ABTS detection solution (50 mM potassium phosphate buffer (pH 5.7), 5% ABTS stock solution (1 mg/mL), 0.05% H_2_O_2_) at RT for 40 min. Absorption was measured at 405 nm using a TriStar^2^ LB 942 multimode plate reader (Berthold Technologies, Bad Wildbad, Germany).

### 4.5. In Vitro Kinase Assay

In vitro kinase assays were conducted in a total volume of 15 µL containing 2 µM APP695 fragment, 10 nM GST-CK1δ, 20 µM CK1δ-derived peptide (in DMSO), 25 mM Tris-HCl (pH 7.0), 10 mM MgCl_2_, 0.1 mM EDTA, 10 µM ATP, and 2 µCi [γ-^32^P]-ATP (Hartmann, Braunschweig, Germany) in dH_2_O at 30 °C for 30 min. The same reaction was performed using the prototypic substrate α-casein (2 µM) as a control. Reactions were stopped by adding 5×SDS loading dye and boiling samples at 95 °C for 5 min. Samples were separated by SDS-PAGE, stained with Coomassie Brilliant Blue, and dried for 45 min under a vacuum. The radioactive signal strength was quantified by Cherenkov counting. Data were normalized to the DMSO control.

### 4.6. Peptide Localization in CK1δ and In Silico Simulation of Peptide Docking to APP

Surface maps of CK1δ highlighting the indicated peptide sequences were generated using PyMOL 2.4 (Schroedinger Inc., New York, NY, USA) and CK1δ structural data deposited in the protein data bank (PDB; entry 6GZM [23]). In order to identify and assess the interaction sites on APP, in silico models predicting the interaction of previously identified binding CK1δ-peptides with either the N-APP/E1 domain (amino acids 18 to 190 of APP695; PDB entry 4PWQ [25]) and/or the E2 domain of APP (amino acids 295 to 500 of APP695; PDB entry 3UMI [26]) were generated using the CABS-dock server for flexible protein–peptide docking (accessed on 3 May 2021) [24]. Images displaying the best-ranked model (according to cluster density) for each docking analysis were created using PyMOL. Secondary structure prediction for human CK1δ (transcription variant 1, UniProt entry P48730) was performed using the PSIPRED server for secondary structure prediction (accessed on 5 May 2021) [28,29].

### 4.7. Maintenance, Passaging, and Lentiviral Transduction of hNPCs

Immortalized hNPCs (ReNcell^®^ VM) were purchased from Merck Millipore (Darmstadt, Germany). Naïve and transduced hNPCs were expanded in proliferation medium consisting of DMEM/F12 medium (Gibco/Life Technologies, Carlsbad, CA, USA) supplemented with 2% (*v/v*) B-27 neural supplement (Gibco/Life Technologies, Carlsbad, CA, USA), 2 µg/µL heparin (Stemcell Technologies Inc., Vancouver, BC, Canada), 100 U/mL penicillin-streptomycin solution (Gibco/Life Technologies, Carlsbad, CA, USA), 2.5 µg/mL amphotericin-B solution (Invitrogen^TM^, Carlsbad, CA, USA), 20 ng/mL human epidermal growth factor (EGF) (Sigma Aldrich, St. Louis, MO, USA), and 20 ng/mL human basic fibroblast growth factor (bFGF) (Reprocell Inc., Glasgow, UK) on Matrigel (Corning Inc., Corning, NY, USA)-coated T25 flasks (Sarstedt, Nümbrecht, Germany), 6-well plates (Sarstedt, Nümbrecht, Germany), or 96-well plates (Sarstedt, Nümbrecht, Germany). Cells were incubated in a humidified atmosphere (5% CO_2_) at 37 °C. The differentiation medium was prepared using the composition of proliferation medium described above without adding EGF and bFGF.

The lentiviral DNA constructs pCSCW-APPSL-IRES-GFP and pCSCW-PSEN1(ΔE9)-IRES-mCherry encoding full-length human APP695 with K670N/M671L/V717I (Swedish and London mutation) and GFP or human PSEN1 (ΔE9) and mCherry, respectively, were kindly provided by Prof. Dr. Doo Kim (Massachusetts General Hospital, Harvard Medical School, Charlestown, MA, USA). Packaging and envelope vectors psPAX2 and pMD2.G for lentiviral particle production were kindly provided by Prof. Dr. Cagatay Günes (Ulm University Hospital, Ulm, Germany). For packaging lentiviruses, 1.8 µg of pSPAX2, 300 ng of pMD2.G and 3 µg of pCSCW-APPSL-IRES-GFP or pCSCW-PSEN1(ΔE9)-IRES-mCherry were co-transfected into 5 × 10^6^ HEK293T cells (obtained from DSMZ, ACC 635, Hannover, Germany) grown on 10 cm culture dishes (Sarstedt, Nümbercht, Germany) by using 30 µL polyethylenimine (Sigma Aldrich, St. Louis, MO, USA). The following day, the lentivirus-containing supernatants were collected, filtered, and stored at −80 °C for later use. For transduction, 80% confluent hNPCs were incubated with viral stocks (approximately 6 × 10^6^ transducing units per mL) supplemented with 10 µg/mL polybrene (Santa Cruz Biotechnology, Dallas, TX, USA) overnight. The next day, cells were washed twice with Dulbecco’s PBS and then supplied with fresh proliferation medium. In order to generate cells expressing both PSEN1(ΔE9)/mCherry and APPSL/GFP, cells transduced with virus particles generated using pCSCW-PSEN1(ΔE9)-IRES-mCherry were infected again with particles generated using pCSCW-APPSL-IRES-GFP. The enrichment of high-expressing transgenic hNPCs via fluorescence-activated cell sorting (FACS) was performed according to Kim et al. [30]. For FACS, the transduced hNPCs were washed with PBS and detached with Accutase^®^ (Sigma Aldrich, St. Louis, MO, USA). Cell pellets were resuspended in ice-cold PBS supplemented with 2% Knockout^TM^ Serum Replacement (Gibco/Life Technologies, Carlsbad, CA, USA) and 2% B-27 to obtain a final concentration of 10^7^ cells/mL and cells were then passed through a cell strainer filter (70 µm, Corning Inc., Corning, NY, USA) prior to sorting. Transduced hNPCs were enriched by using a FACSAria cell sorter (BD, Franklin Lakes, NJ, USA) located at the Core Facility Cytometry at Ulm University. Transduced cells were sorted by using GFP and/or mCherry channels. Sorted cells were maintained and expanded in proliferation medium.

### 4.8. 3D Cell Culture of hNPCs

3D culture was performed as described previously [30]: for 3D cultivation, transduced and enriched hNPCs were resuspended in ice-cold differentiation medium and kept on ice. For thick-layer 3D culture, Matrigel was added to ice-cold cell suspensions (1:2 dilution rate) to obtain a final concentration of 1 × 10^7^ cells/mL. Three hundred µL of the cell/Matrigel suspension was immediately transferred into tissue culture inserts in 24-well plates (Corning Inc., Corning, NY, USA) using pre-chilled pipette tips. For thin-layer 3D culture, additional ice-cold differentiation medium was added to the cold cell/Matrigel suspension (1:10 total dilution rate) to a final concentration of 1 × 10^6^ cells/mL. The plating volume was 600 µL/well for 24-well plates. The following day, 1 mL fresh differentiation medium per well was added.

### 4.9. Treatment of 2D and 3D Cultures with CK1δ-Derived Peptides and SMIs

Depending on the given experiment, naïve or transduced hNPCs were differentiated for six days and treated with the indicated peptides or SMIs for up to six weeks. For the analysis of peptide cell entry, 2D-differentiated naïve hNPCs were grown onto Matrigel-coated glass slides in 6-well plates at a concentration of 1.5 × 10^5^ cells/mL. Treatment was performed by incubating cells with 1 µM CK1δ-derived peptides for 24 h.

In order to treat transduced hNPCs differentiated in 3D cultures (either thin- or thick-layer culture), 1 µM of CK1δ-derived peptides and 10 µM β-secretase inhibitor IV (Cayman Chemical, Ann Arbor, MI, USA [43]) or DMSO as the solvent control were added. In long-term treatment schemes, media were exchanged every second day.

### 4.10. Immunofluorescence Staining

After treatment was finished, cells were washed three times (2D culture) or only briefly (thin-layer 3D culture) with 1× PBS. Washed cells were fixed with 4% (*v/v*) paraformaldehyde in 1× PEM buffer (80 mM PIPES (pH 6.8), 1 mM EGTA, 5 mM MgCl_2_) at 4 °C for 20 min (2D) or overnight (3D) followed by permeabilization by 0.3% (*v/v*) Triton-X 100 in 1× PEM buffer for 5 min (2D) or 1 h (3D) at RT. After brief washing with 1× PEM, cells were blocked with 5% (*w/v*) BSA in 1× PEM for 30 min (2D) or 8 h (3D) at RT. Thereafter, cells were washed three times in 1× PEM and incubated with anti-β-III-tubulin antibody (MAB1637, Merck Millipore, Darmstadt, Germany; 1:500 in 1× PEM) or anti-β-amyloid antibody (6E10, BioLegend, San Diego, CA, USA; 1:500 in blocking solution) at 4 °C overnight. For 3D cultures, antibody incubation was performed under gentle rocking. Cells were washed three (2D) or five (3D) times with 1× PEM and subsequently incubated with DyLight 488 anti-mouse antibody (Thermo Fisher Scientific Inc., Waltham, MA, USA; 1:200) or DyLight 350 anti-mouse antibody (Thermo Fisher Scientific Inc., Waltham, MA, USA; 1:500) at RT for 40 min (2D) or 5 h (3D). Then, cells were washed three (2D) or five (3D) times with 1× PEM, and 2D cultures were additionally stained with TRITC-streptavidin (Jackson ImmunoResearch Europe Ltd., Cambridgeshire, UK; 1:200) at RT for 30 min and 0.1 µg/mL DAPI (Sigma Aldrich, St. Louis, MO, USA) at RT for 5 min. Finally, washed cells were mounted with ProLong^TM^ Glass Antifade Mountant (Invitrogen^TM^, Carlsbad, CA, USA). Fluorescence images were captured using an Olympus IX81 microscope and attached XM10 camera (Olympus Europa, Hamburg, Germany) at 10× magnification and analyzed with the Olympus software cellSens Dimensions 2.3 (Build 18,987, Olympus Europa, Hamburg, Germany). Within each experiment, imaging settings and acquisitions were maintained constant between different samples. Cells and peptide accumulations were counted manually. The peptide uptake ratio was calculated as the number of detected peptide accumulations per cell (the number of peptide accumulations divided by the total cell count).

### 4.11. Quantitative Analysis of Aβ Levels and Monitoring of Cytotoxic Effects

Levels of Aβ_1-42_ were measured by Quantikine^®^ human amyloid β (amino acids 1–42) immunoassay (R&D Systems, Minneapolis, MN, USA). Therefore, transduced differentiated hNPCs were grown in thick-layer 3D culture as described above, and conditioned media were collected and diluted 1:2 prior to use in the immunoassay by using dilution buffer provided by the manufacturer. ELISA signals were read using a Tecan Spark 10M microplate reader (Tecan, Männedorf, Switzerland). The status of 3D cultures was constantly monitored by performing LDH release assay to exclude cytotoxic effects mediated by the respective treatment (CytoTox-ONE Homogenous Membrane Integrity Assay, Promega, Fitchburg, MA, USA). Results obtained from the cytotoxicity assay were expressed as a fold change of cytotoxicity compared to the untreated control at 2 days.

### 4.12. Statistical Analysis

GraphPad Prism 8.0 (GraphPad Software, La Jolla, CA, USA) was used to perform statistical analyses. Results were presented as the mean of experiments performed as technical triplicates with error bars (standard deviation). Based on the underlying hypotheses of the specific experiments, experimental data was compared by using a one-tailed Student’s *t*-test. Differences between groups were considered statistically significant for *p* ≤ 0.05 (shown as * for *p* ≤ 0.05, ** for *p* ≤ 0.01, *** for *p* ≤ 0.001, or **** *p* ≤ 0.0001).

## 5. Conclusions

With the aim to identify peptide therapeutics for the treatment of AD, the potential of CK1δ-derived peptides was successfully tested biochemically and in cell culture. Selected peptides (i) demonstrated APP695-selective competitive inhibition of CK1δ-mediated phosphorylation, (ii) showed no long-term cytotoxic effects in cell culture, and (iii) significantly reduced the levels of secreted Aβ and amyloid plaque formation in a cell culture model for AD. By achieving these goals, the tested peptides provided initial proof of their therapeutic potential, which now has to be extended by optimization approaches and further validation using more sophisticated model systems in vitro and in vivo.

## Data Availability

All data is contained within the article and Supplementary Materials.

## Supplementary Materials

The following are available online at https://www.mdpi.com/article/10.3390/ijms22126423/s1, Figure S1: Schematic overview of the CK1δ-derived peptide library with indicated peptide sequences and functional domains, Figure S2: Initial ELISA screenings indicating potential binding of CK1δ-derived peptide to APP695 fragments, Figure S3: CK1δ-mediated phosphorylation of APP695 fragments, Figure S4: Effect of CK1δ-derived peptides on CK1δ-mediated phosphorylation of α-casein as control, Figure S5: Peptide-protein docking models simulating interactions of CK1δ-derived peptides with the N-APP/E1 protein domain of APP, Figure S6: Peptide-protein docking models simulating interactions of CK1δ-derived peptides with the E2 protein domain of APP, Figure S7: Cell viability monitoring of 3D cell cultures treated with CK1δ-derived peptides, Table S1: Peptide-protein docking data for simulating interactions of CK1δ-derived peptides with APP.
