# Supplementary material for: CK1δ-Derived Peptides as Novel Tools Inhibiting the Interactions between CK1δ and APP695 to Modulate the Pathogenic Metabolism of APP
Source: Int J Mol Sci. 2021 Jun 15;22(12):6423. doi: 10.3390/ijms22126423 (PMC8232658; doi:10.3390/ijms22126423)
Supplement: Supplementary file 1 [file ijms-22-06423-s001.zip › ijms-1258071-supplementary.pdf]

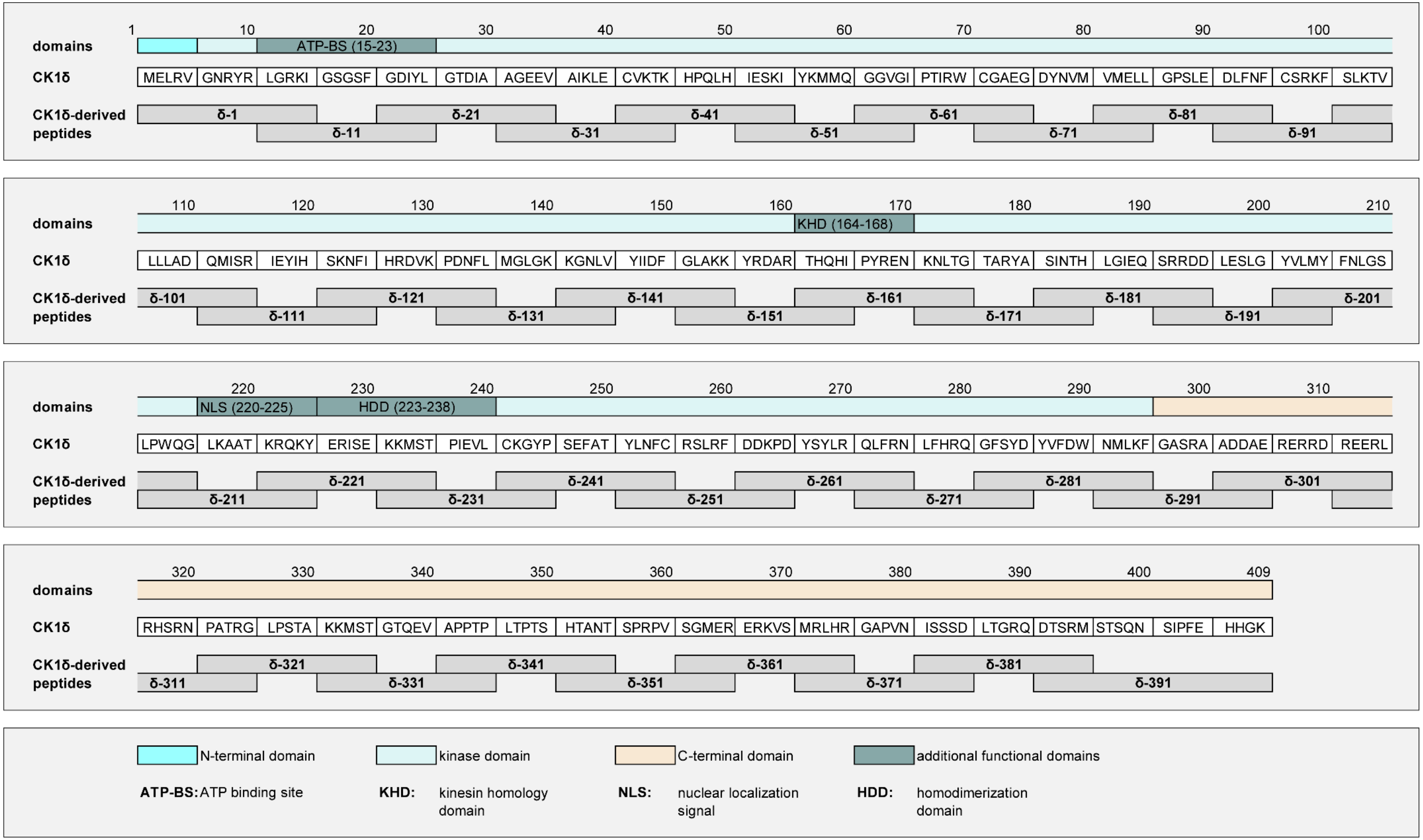

Supplementary Figure S1: Schematic overview of the CK1δ-derived peptide library with indicated peptide sequences and functional domains.

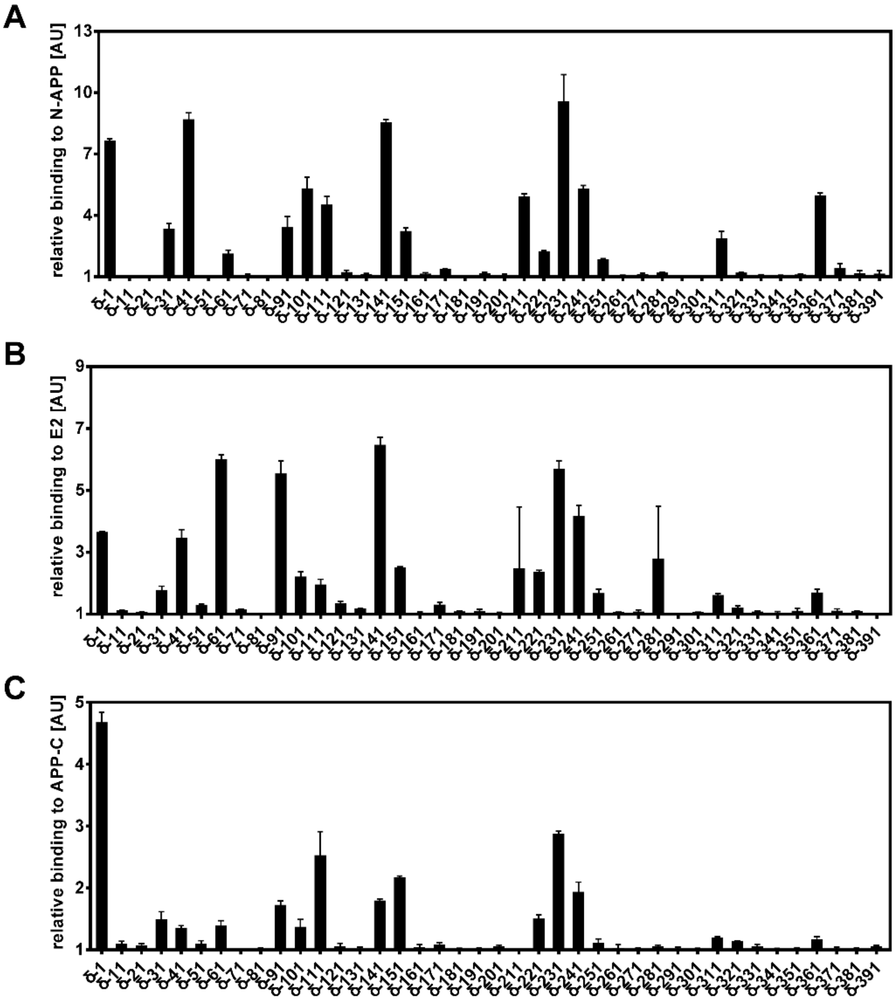

Supplementary Figure S2: Initial ELISA screenings indicating potential binding of CK1δ-derived peptide to APP695 fragments. Relative binding of CK1δ-derived peptides to coated **(A)** N-APP, **(B)** E2 or **(C)** APP-C compared to control (n = 3).

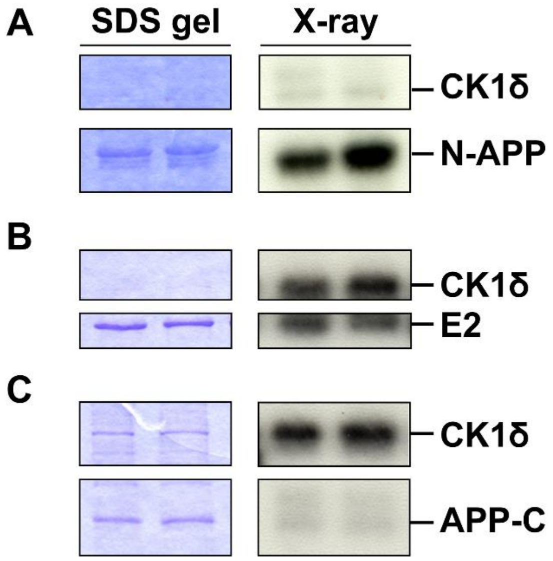

Supplementary Figure S3: CK1δ–mediated phosphorylation of APP695 fragments. *In vitro* kinase assay reactions containing GST-CK1δ, [γ-32P]-ATP and purified **(A)** N-APP, **(B)** E2 or **(C)** APP-C were separated by SDS-PAGE and proteins were visualized by staining with Coomassie (left; SDS gel). Phosphorylation of APP695 fragments as well as kinase autophosphorylation was indicated by incorporation of radioactively labelled 32P and visualized by autoradiography (right; X-ray). SDS: sodium dodecyl sulphate.

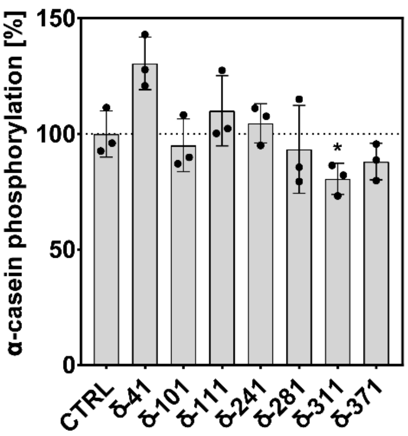

Supplementary Figure S4: Effect of CK1δ-derived peptides on CK1δ-mediated phosphorylation of α-casein as control. In order to determine general and substrate-unspecific inhibition of CK1δ by CK1δ-derived peptides, *in vitro* kinase assays were performed in the presence and absence (DMSO) of 20 μM of the indicated CK1δ-derived peptides using GST-CK1δ as kinase and α-casein as substrate (n = 3). Statistical analysis was performed using one-tailed t-test shown as * for $p \leq 0.05$.

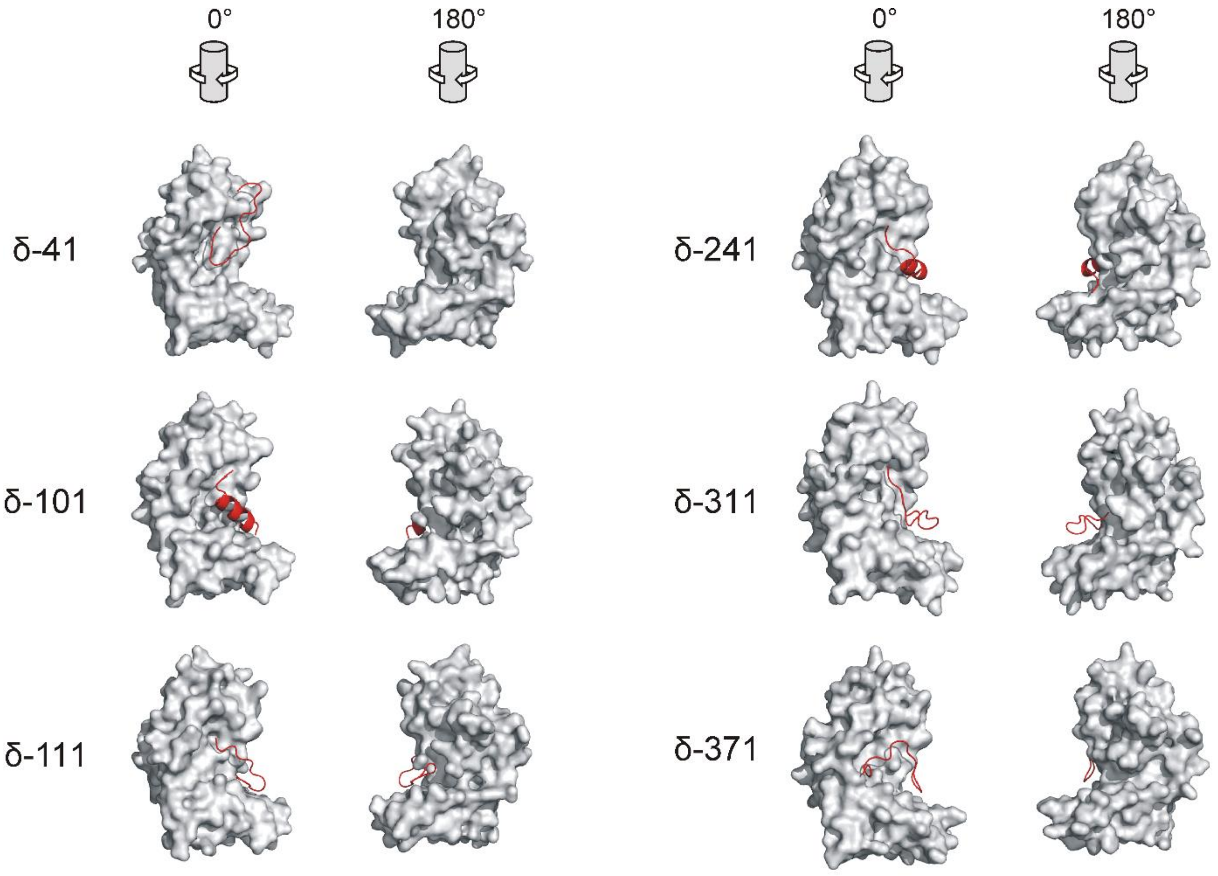

Supplementary Figure S5: Peptide-protein docking models simulating interactions of CK1δ-derived peptides with the N-APP/E1 protein domain of APP. CK1δ-derived peptides previously identified to bind and inhibit phosphorylation of APP695 protein fragments were used for *in silico* docking experiments using a protein structure encompassing the N-APP/E1 domain of APP (4PWQ [25]). Docking simulation was performed by CABS-dock server for flexible protein-peptide docking and the best-ranked model (according to cluster density) for each docking analysis is displayed in original view (0°) and after rotation by 180°. Peptide structures are shown in red.

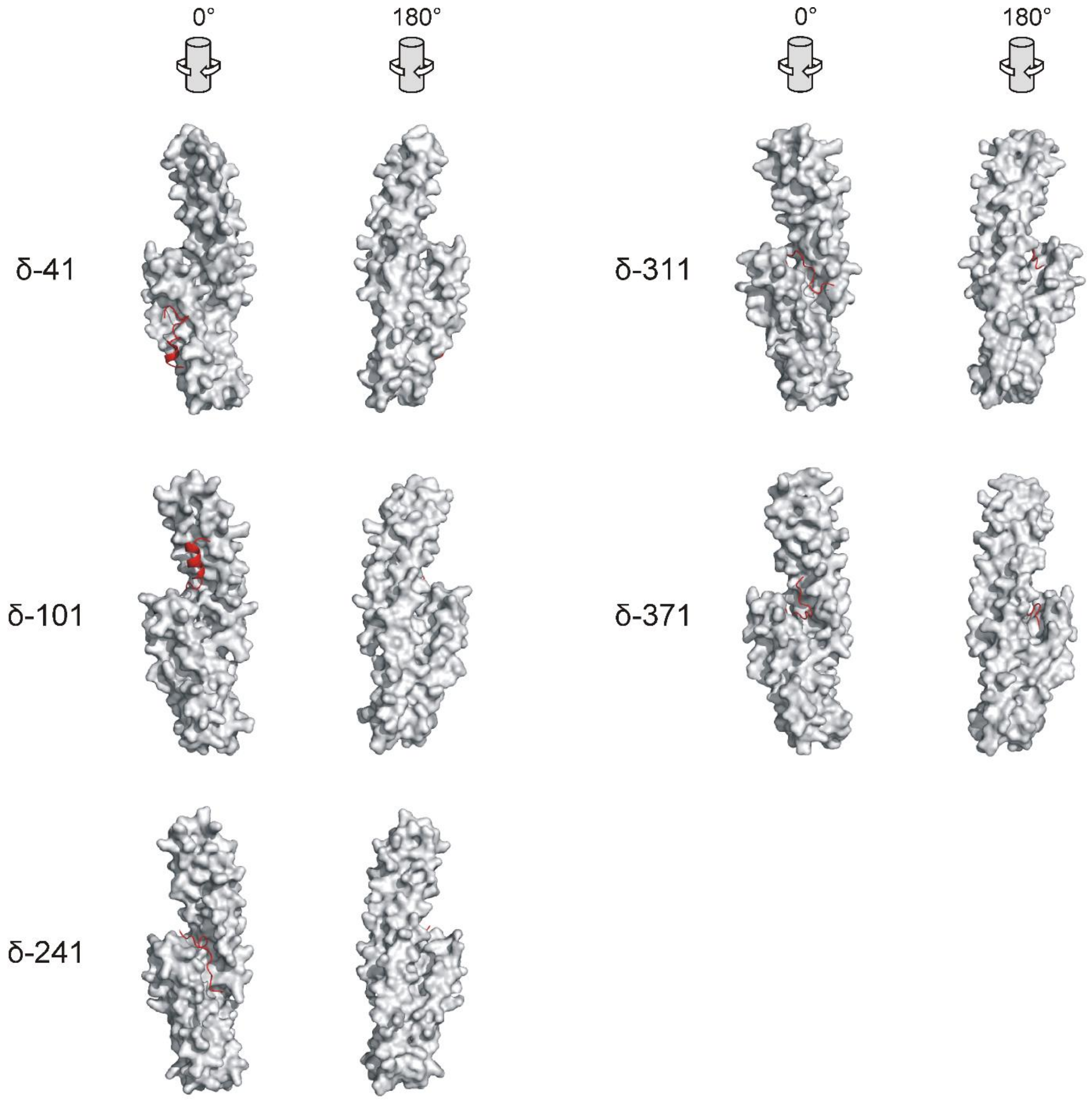

Supplementary Figure S6: Peptide-protein docking models simulating interactions of CK1δ-derived peptides with the E2 protein domain of APP. CK1δ-derived peptides previously identified to bind and inhibit phosphorylation of APP695 protein fragments were used for *in silico* docking experiments using a protein structure encompassing the E2 domain of APP (3UMI [26]). Docking simulation was performed by CABS-dock server for flexible protein-peptide docking and the best-ranked model (according to cluster density) for each docking analysis is displayed in original view (0°) and after rotation by 180°. Peptide structures are shown in red.

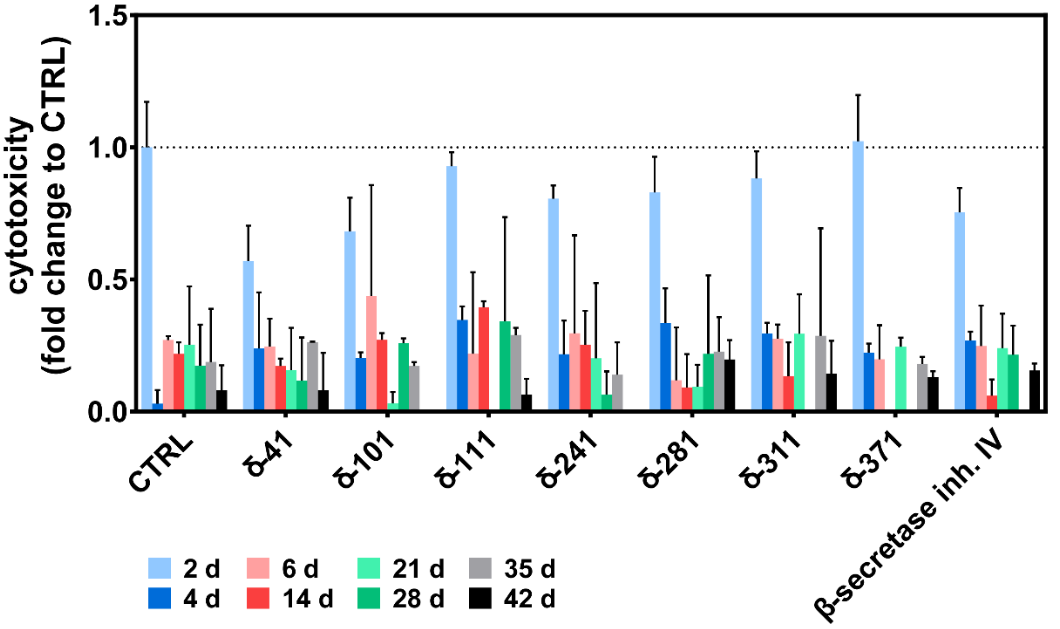

Supplementary Figure S7: Cell viability monitoring of 3D cell cultures treated with CK1δ-derived peptides. Transduced and differentiated hNPCs were treated with 1 µM of the indicated CK1δ-derived peptides or 10 µM of β-secretase inhibitor IV. Routine monitoring of cell viability of thick-layer 3D cultures was performed by LDH release assay during the entire observation period of six weeks (42 d). Cell viability is displayed as fold change using value determined for control cell culture (CTRL after 2 d) treated with DMSO as reference value (n = 3).

Supplementary Table S1: Peptide-protein docking data for simulating interactions of CK1δ-derived peptides with APP. CK1δ-derived peptides previously identified to bind and inhibit phosphorylation of APP695 protein fragments were used for *in silico* docking experiments using a protein structure encompassing **(A)** the N-APP/E1 domain (4PWQ [25]) or **(B)** the E2 domain of APP (3UMI [26]). Docking simulation was performed by CABS-dock server for flexible protein-peptide docking and cluster density as well as average RMSD values for the best-ranked model are shown in each case. Structural information for the corresponding peptide sequence in full-length CK1δ is indicated according to PSIPRED-based prediction of secondary structure features [28,29] using the protein sequence of human CK1δ (transcription variant 1; UniProt entry P48730). C: coil (residues in no further specified conformation); E: extended strand in parallel and/or anti-parallel β-sheet conformation; H: α-helix; RMSD: root mean square deviation.

A

Peptide	peptide sequence	peptide structure in full-length CK1δ	cluster density	average RMSD
δ-41	CVKTK HPQLH IESKI	ECCCCCHHHHHHHHH	27.3162	7.54132
δ-101	SLKTV LLLAD QMISR	CHHHHHHHHHHHHHH	39.5634	2.88145
δ-111	QMISR IEYIH SKNFI	HHHHHHHHHHCCCC	37.3641	3.45252
δ-241	CKGYP SEFAT YLNFC	HCCCCHHHHHHHHHH	44.3991	4.23432
δ-311	REERL RHSRN PATRG	HHHHHHHHCCCCCCC	28.6284	4.15671
δ-371	MRLHR GAPVN ISSSD	CCCCCCCCCCCCHHH	28.5258	3.96132

B

Peptide	peptide sequence	peptide structure in full-length CK1δ	cluster density	average RMSD
δ-41	CVKTK HPQLH IESKI	ECCCCCHHHHHHHHH	28.9064	5.3973
δ-101	DLFNF CSRKF SLKTV	CHHHHHHHHHHHHHH	31.6405	3.79261
δ-241	CKGYP SEFAT YLNFC	HCCCCHHHHHHHHHH	147.427	0.678303
δ-311	REERL RHSRN PATRG	HHHHHHHHCCCCCCC	48.438	2.22966
δ-281	GFSYD YVFDW NMLKF	CCCCCCCCCCHHHCC	41.7527	3.61654
